# A review of multi-omics integration techniques across five machine learning method families

**DOI:** 10.1093/bioadv/vbag108

**Published:** 2026-04-15

**Authors:** Adedayo Olowolayemo, Amina Souag, Konstantinos Sirlantzis, Scott Turner, Cornelia Wilson

**Affiliations:** Department of Computing, AI and Cybersecurity, Canterbury Christ Church University, CT1 1QU, Canterbury, UK; Department of Computing, AI and Cybersecurity, Canterbury Christ Church University, CT1 1QU, Canterbury, UK; Department of Computing, AI and Cybersecurity, Canterbury Christ Church University, CT1 1QU, Canterbury, UK; Department of Computing, AI and Cybersecurity, Canterbury Christ Church University, CT1 1QU, Canterbury, UK; Department of Natural Sciences, Canterbury Christ Church University, CT1 1QU, Canterbury, UK

## Abstract

**Motivation:**

Multi-omics integration methods are now common in cancer studies, but results remain sensitive to design choices, including when fusion occurs, what is fused, and how missingness is handled. As a result, it is difficult to compare studies and determine which integration choices are most reliable for cross-cohort cancer analyses.

**Results:**

From a PRISMA-guided review of 30 studies (2020–2025), we find that graph-based or hybrid pipelines dominate, with deep learning as the next most common family, and survival prediction as the main use case. Method families tend to align with the task and time of fusion; graph-hybrid approaches favour early- to intermediate-stage fusion, while deep learning spans the three stages of fusion. Across studies, three recurring trade-offs emerge: early-intermediate fusion can stabilize high-dimensional inputs but is sensitive to modality imbalance; shared latent-space designs better preserve partially observed samples; and late fusion supports more stable subtype structure but makes feature attribution less direct. The main message is that integration works best when fusion choices match the data’s noise, sparsity, and missingness, and when interpretability is built into the architecture rather than added later.

## Introduction

Cancer is fundamentally a disease of uncontrolled cell proliferation caused by regulatory failures (Brown *et al*. 2023). Since tumours in different organs often share molecular features, researchers are increasingly using pan-cancer analysis to study these diverse types within a single framework (Hoadley *et al*. 2018, Aaltonen *et al*. 2020). The goal is to find common biological patterns that might be missed in single-tumour studies, while still accounting for differences between cancer types (Weinstein *et al*. 2013, Geffen *et al*. 2023). Multi-omics profiling extends this approach by examining genomes, transcriptomes, epigenomes, and proteomes (Savage *et al*. 2024).

While no single method captures the entire biological system, integrating these layers can reveal connections that are not visible in isolation, such as how DNA mutations affect RNA expression and protein levels (Argelaguet *et al*. 2018, Zhang *et al*. 2022, Flores *et al*. 2023). The main challenge here is methodological: the way we combine information, whether through raw features, shared representations, or sample relationships, determines which biological signals we can detect.

This design process is often limited by two practical issues. First, data is often incomplete, with many tumours missing specific omics layers (Flores *et al*. 2023, Baião *et al*. 2025). Second, the data types vary widely in their statistical properties, from sparse mutation data to dense expression profiles. Simple methods, like concatenating all features at the start, run the risk of letting one data type dominate the analysis (Picard *et al*. 2021, Leng *et al*. 2022). Generally, integration strategies fall into three categories: early fusion (concatenating features), intermediate fusion (estimating shared representations), and late fusion (combining sample relationships or graphs) (Bersanelli *et al*. 2016). These categories align with the five method families we assess: linear projection, kernel-based, probabilistic, deep learning, and graph-based/hybrid methods (Liu *et al*. 2024, Nikolaou *et al*. 2025).

Although many reviews exist, most are limited by a narrow scope (Baião *et al*. 2025). Current taxonomies, such as those by Vahabi and Michailidis (2022) and Abdelaziz *et al*. (2024), often classify methods by their statistical or machine-learning basis, but tend to overlook the biological reasoning behind them. Similarly, the literature is often divided into technical benchmarks, such as Leng *et al*. (2022), which treat integration as a “black box,” or application-focused reviews like Acharya and Mukhopadhyay (2024) that prioritize clinical workflows over model architecture.

Consequently, while the field has advanced, there remains an opportunity to more explicitly frame multi-omics integration as a conceptual architectural decision, particularly in pan-cancer contexts where distinguishing universal drivers from tissue-specific noise is critical. In this review, we analyze these five method families through a structural lens. Rather than focusing solely on tool selection, we synthesize these families to clarify the logic behind fusion, examining signal assumptions, how missing data is handled, and interpretability. Our aim is to provide a unified framework for understanding how different integration choices influence cancer and pan-cancer research.

## Methods

This systematic review followed PRISMA 2020 guidelines to ensure transparency and rigour. As a methodological review emphasizing computational integration strategies rather than clinical outcomes, it was not preregistered (Booth *et al*. 2012, Page *et al*. 2021).

**Figure 1:**
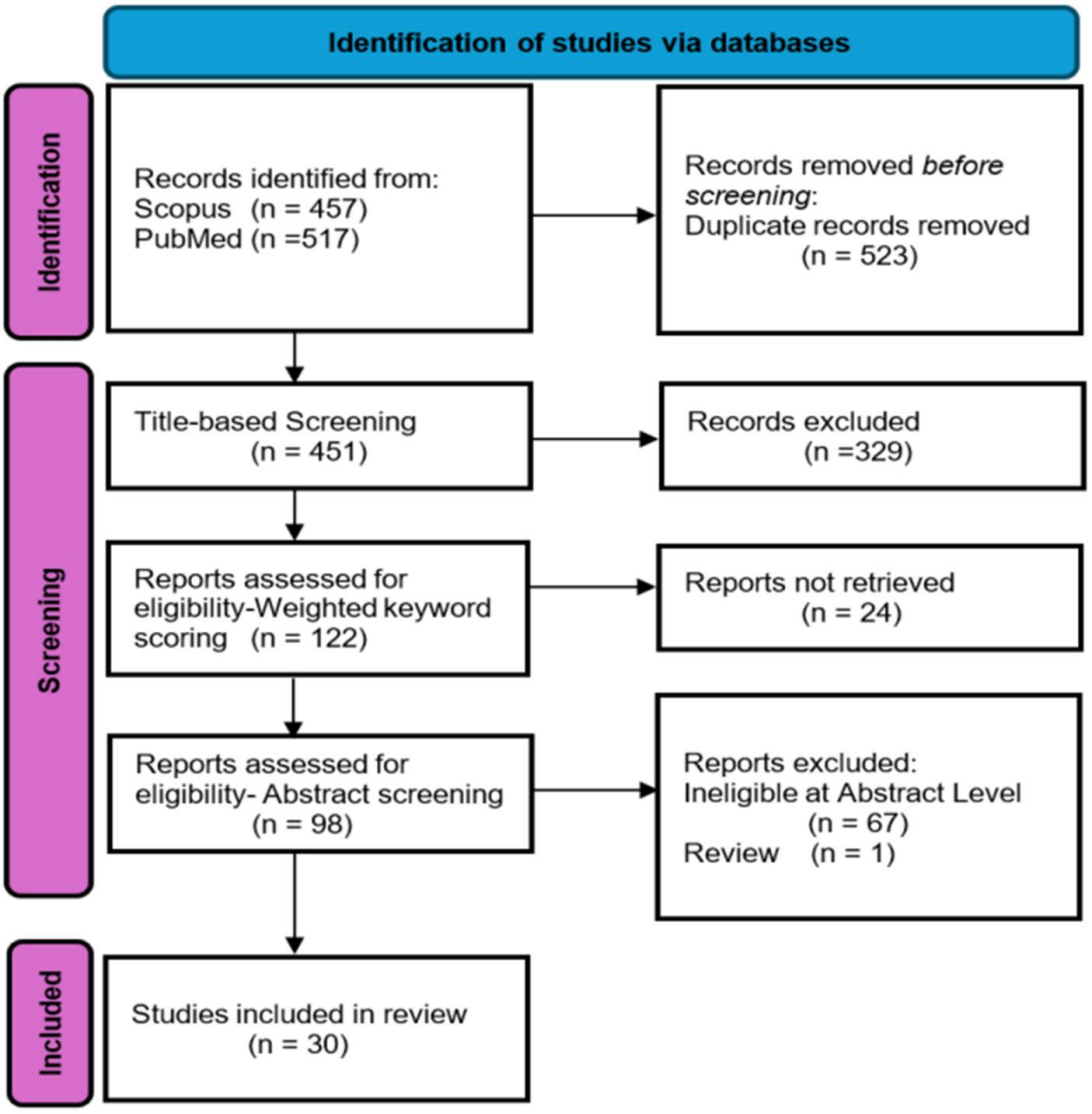
PRISMA 2020 flow diagram for study selection. Records were identified from Scopus (n=457) and PubMed (n=517); after removal of 523 duplicate records, 451 unique records underwent title-based screening. Following weighted keyword scoring (122 records assessed) and abstract screening (98 records assessed), 30 studies were included in the final review. Records excluded at abstract screening were ineligible at abstract level (n=67) or were review articles (n=1).

PubMed and Scopus databases were searched using a structured approach combining multi-omics integration, cancer-related terms, pan-cancer indicators, and computational methods. Database-specific operators were applied, yielding 451 unique records after de-duplication (Figure 1).

Relevance was prioritised via a weighted keyword scoring scheme applied to normalised titles. Weights were assigned to core-scope terms (3), domain/method terms (2), and general modelling terms (1). Records with a cumulative score ≤2 were excluded, while those scoring ≤4 were retained only if containing at least one core-scope keyword. This process yielded 98 articles for abstract screening.

Abstract screening applied three inclusion criteria: (1) integration of at least two omics modalities; (2) use of an explicit integration method (e.g., MOFA, iCluster, SNF, CCA, VAE, GNN, MKL) or unambiguous integrative phrasing; and (3) a defined pan-cancer scope. Following manual verification of omics layers and named methods, 30 studies were selected for full-text review.

## Fusion logic and method families

Multi-omics integration in cancer research addresses the systemic nature of tumour biology, capturing coordinated molecular processes beyond tissue-specific boundaries (Hanahan 2022, Mambetsariev *et al*. 2023, Swanton *et al*. 2024). At the center of this is integration, a core modelling decision determining signal visibility. Fusion strategies generally fall into three paradigms: simple aggregation, correlation-based integration, and structure-driven fusion (Argelaguet *et al*. 2020, Aldoseri *et al*. 2023, Ballard *et al*. 2024).

Simply aggregating data from various omics risks masking signals under technical noise (Lan *et al*. 2024). While correlation-based methods, such as Canonical Correlation Analysis (CCA) do model the inter-layer relationships but often lack interpretability (Jiang *et al*. 2023a), structure-driven integration—the area we explored in this study—embeds fusion into the architecture via shared latent spaces, kernels, or biological networks (Cao *et al*. 2024, Schuster *et al*. 2024, Creux *et al*. 2025).

While fusion stage provides a useful organizing lens in this review, not all included studies report integration timing in a form that permits stage assignment; accordingly, Table 3 presents only the classifiable subset alongside method family, modalities, supervision, and task.

**Table 3:** Study-level design summary of included studies stratified by fusion stage into early, intermediate, and late.

Part 1: Early and Intermediate fusion						
Fusion Stage	Ref	Method Family	Cancer Types	Omics Modalities	Supervision Type	Task
Early	Bai and Zhang (2023)	Hybrid	33 TCGA cancer types	Transcriptomics: mRNA; Transcriptomics: miRNA/lncRNA	Supervised	Pan-cancer classification
	Wang *et al.* (2024)	Deep learning	Training: Pan-cancer (UCSC Xena, 36 types). Testing: BLCA, NSCLC, SKCM	Transcriptomics: bulk RNA (TPM) for prediction; scRNA used for feature discovery	Supervised	Predict response to anti-PD-1/PD-L1 therapy from bulk RNA using multi-level feature integration
	Li *et al.* (2022)	Linear Projection-Based, Probabilistic, and Deep Learning	TCGA Pan-Cancer (33 types)	Genomics: mutation-derived features; Genomics: CNV; Epigenomics: histone marks & DNA methylation; Phenotypic: CRISPR dependency, expression Z-scores, VEST	Supervised (binary: driver vs non-driver)	Quantify multi-omics feature contributions and identify effective combinations for driver-gene detection
	Moravveji *et al.* (2022)	Probabilistic	ESCA; CHOL; STAD; COAD; PAAD; LIHC; READ	Transcriptomics: RNA-seq; Epigenomics: DNA methylation (450k); Genomics: CNV (Affymetrix SNP 6.0)	Unsupervised for integration; supervised RF for feature importance	Detect combinatorial impacts of expression, methylation, and CNV on GI cancers; identify key subnetworks
	Guo *et al.* (2022)	Deep learning + statistical survival	TCGA pan-cancer (33 types)	mRNA; miRNA (incl. isomiRs); lncRNA; circRNA; DNA methylation (450k/27k); CNV (GISTIC2)	Self-supervised (DAE) + supervised survival	Reveal multi-omics features and RNA cross-talks of the Notch pathway; assess prognostic ability
Intermediate	Chierici *et al.* (2020)	Graph	BRCA; AML; KIRC (clear cell RCC)	Transcriptomics: mRNA; Genomics: CNV; Proteomics; Epigenomics: DNA methylation; miRNA/lncRNA	Supervised	Improve predictive performance and compactness of multi-omics signatures
	Jiang *et al.* (2024)	Graph	BLCA; BRCA; CESC; COAD; ESCA; HNSC; PAAD; PCPG; PRAD; SKCM; THCA	Transcriptomics: mRNA; Epigenomics: DNA methylation; Genomics: Mutation	Supervised	Metastasis probability prediction (pan-cancer)
	Tan *et al.* (2020)	Deep	TCGA pan-cancer (∼30 types)	DNA methylation; miRNA-Seq; RNA-Seq; RPPA proteomics	Supervised (binary classification)	Predict clinical outcome endpoints (OS, DSS, PFI, DFI)
	Du *et al.* (2025)	Graph/Hybrid	CRC (primary); lung; breast; ovarian (extensions)	Transcriptomics: single-cell; Transcriptomics: spatial; Proteomics: spatial	Unsupervised	Identify boundary-specific intercellular regulatory axes in CRC using spatial multi-omics
	Chen *et al.* (2024)	Probabilistic	TCGA pan-cancer (33 types) with BLCA deep-dive	Transcriptomics: bulk RNA; Genomics: SNV; CNV; Epigenomics: DNA methylation/stemness; Proteomics: IHC (HPA)	Unsupervised + survival regression (Cox)	Assess prognostic and immunological significance of HDAC family across cancers
	L. Li *et al*. (2021)	NA	ACC, BLCA, BRCA, COAD, ESCA, HNSC, KICH, KIRC, KIRP, LGG, LIHC, LUSC, OV, PAAD, PCPG, SARC, SKCM, STAD, THCA, THYM, UCEC	Somatic mutations (WES-derived TMB); mRNA expression; DNA methylation (450k/27k)	Unsupervised grouping + supervised survival associations	Characterize how TMB relates to prognosis and multi-omic features across cancers
	Yang and Li (2025)	Probabilistic (linear projection-based)	LUAD; COAD; LUSC; SARC; BLCA; BRCA; HNSC; KIRC; LGG; LIHC (TCGA)	Bulk mRNA expression; DNA methylation (450k); miRNA expression	Unsupervised	Integrate multi-modal data with missing entries for clustering and downstream analyses

Part 2: Late fusion						
Fusion Stage	Ref	Method Family	Cancer Types	Omics Modalities	Supervision Type	Task
Late	Chen *et al.* (2022)	Deep learning	BLCA; BRCA; COADREAD; HNSC; KIRC; KIRP; LGG; LIHC; LUAD; LUSC; PAAD; SKCM; STAD; UCEC	Pathology/Imaging; Genomics: Mutation; CNV; Transcriptomics: mRNA	Supervised	Improve pan-cancer survival risk stratification via multimodal fusion
	Bhattacharyya *et al.* (2024)	Probabilistic/Bayesian	TCGA pan-cancer (14 cancers: pan-gynecological, pan-gastrointestinal, pan-squamous, pan-kidney)	Transcriptomics: mRNA; Proteomics (RPPA); Genomics: CNV; Epigenomics: DNA methylation	Supervised (outcome regression: survival/continuous stemness)	Improve detection of outcome-associated proteogenomic biomarkers via functional mechanistic evidence
	Bhattacharyya *et al.* (2022)	Probabilistic/Bayesian	Breast (BRCA); Ovary (OV); Uterus (UCS); cell-line lineages: breast, ovary, uterus	Transcriptomics: mRNA; Genomics: copy-number; Epigenomics: DNA methylation; Outcome: drug response	Supervised (outcome regression); mechanistic evidence via Bayesian model comparison	Synthesize cross-system multi-omics evidence to improve detection of functional drivers and drug associations
	Mohammed *et al.* (2023)	Deep learning + Gradient boosting + Federated learning	Skin cancer; Lung cancer; Bone marrow cancer; Lymphoma	Transcriptomics: mRNA; Non-coding RNA: miRNA; Epigenomics: DNA methylation	Supervised at decision layer (XGBoost); auto-encoder self-supervised pretraining	Improve accuracy, reduce processing delay, enhance security for multi-omics multi-cancer detection
	Paczkowska *et al.* (2020)	Probabilistic	PCAWG 38 tumour types; METABRIC breast (HER2+, basal-like, luminal A/B)	Somatic mutations (coding & non-coding: UTRs, promoters, enhancers); CNV; Bulk mRNA (via ISOpure); ChIP-seq TF binding (YAP)	Unsupervised (enrichment/statistical)	Discover pathways enriched across multiple omics by statistically fusing evidence and annotating modality contributions
	Zhang *et al.* (2025)	Probabilistic	TCGA pan-cancer (32 major types)	Transcriptomics (RNA-seq); Proteomics (RPPA); CNV (SNP array); Somatic mutations (exome); DNA methylation	Supervised	Systematically catalog pan-cancer multi-omic correlates of OS transcending tumour lineage
	Scott *et al.* (2021)	Probabilistic	TCGA 23 cancers for methylation; expression meta-analysis across 5 cancers; lung (IHC); pancreatic (serum ELISA)	DNA methylation; bulk transcriptomics; single-cell RNA-seq; proteomics (IHC) + serum protein (ELISA)	Unsupervised meta-analysis + supervised survival/ROC	Identify and validate a robust pan-cancer early biomarker (KRT8) across molecular modalities
	Huang and Kuan (2024)	Kernel based	TCGA pan-cancer subset (12 tissues)	Copy number; DNA methylation; mRNA expression; microRNA expression; protein expression	Unsupervised	Improve integrative clustering accuracy and efficiency across heterogeneous multimodal datasets

*Abbreviations:* TCGA, The Cancer Genome Atlas; RPPA, reverse-phase protein array; WES, whole-exome sequencing; CNV, copy-number variation; SNV, single-nucleotide variant; IHC, immunohistochemistry; scRNA, single-cell RNA; OS/DSS/PFI/DFI, overall/disease-specific/progression-free/disease-free survival.

### Fusion strategy overview

**Figure 2:**
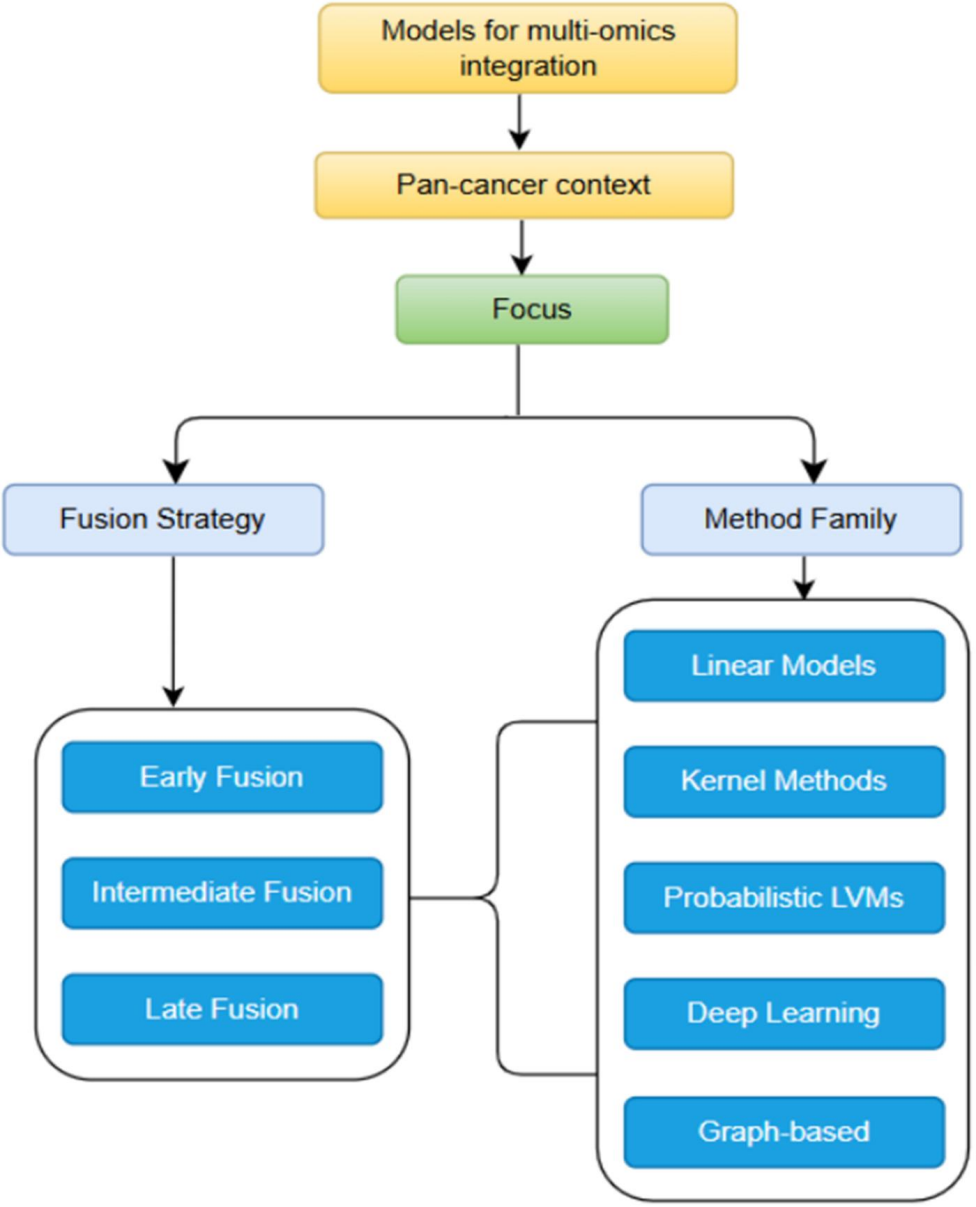
Conceptual structure of this review article. The review focuses on multi-omics integration models in a pan-cancer context, organized along two analytical axes: fusion strategy (early, intermediate, and late fusion) and method family (linear models, kernel methods, probabilistic latent variable models, deep learning, and graph-based approaches). The mapping between fusion strategies and method families forms the structural lens of the review.

Fusion strategies are categorized by timing: early, intermediate, or late (Sharifi-Noghabi *et al*. 2019, Carrillo-Perez *et al*. 2022, Benkirane *et al*. 2023). This distinction often shapes the model’s architecture and biological assumptions. The current review is framed in line with this (Figure 2).

### Early fusion

Early fusion concatenates raw features from multiple modalities into a single input matrix before transformation (Cai *et al*. 2022, Stahlschmidt *et al*. 2022). If $X^{\left(v\right)}$ denotes the data matrix from *v* omics, where *N* is the number of samples and $D_{v}$ is the number of features in modality *v*, the combined matrix is:


(1) $$X_{\text{early}}=[X^{\left(1\right)},X^{\left(2\right)},\ldots ,X^{\left(v\right)}],$$

Where $X_{\text{early}}\in R^{N\times (D_{1}+D_{2}+\cdots +D_{V})}$.

This unified representation (Barnum *et al*. 2020) allows machine learning models to detect cross-modal interactions (Flores *et al*. 2023, Valous *et al*. 2024). However, heterogeneity in scale and sparsity can cause one modality to dominate variance (Garali *et al*. 2018, Hernández-Lemus and Ochoa 2024). Additionally, it assumes complete data availability, necessitating imputation or exclusion when layers are missing (Baker *et al*. 2024).

### Intermediate fusion

Intermediate fusion processes each omics layer independently before combining representations (Beaude *et al*. 2025). Each modality *v* passes through a specific encoder $f^{\left(v\right)}$, such as a neural network or a kernel, to extract a latent embedding (Singh *et al*. 2019, Briscik *et al*. 2024):


(2) $$z^{\left(v\right)}=f^{\left(v\right)}\left(x^{\left(v\right)}\right),$$

Where $x^{\left(v\right)}$ is the *i*-th sample input and $z^{\left(v\right)}\in R^{K_{v}}$.

These embeddings are fused in a shared space via a fusion function $Z=\text{Fusion}(z^{\left(1\right)},z^{\left(2\right)},\ldots ,z^{\left(V\right)})$ (Argelaguet *et al*. 2020, Ashuach *et al*. 2023). This strategy preserves modality-specific structure while enabling joint analysis (Boulahia *et al*. 2021, Stahlschmidt *et al*. 2022).

### Late fusion

Late fusion occurs at the prediction stage, where separate models are trained on each omics layer (Steyaert *et al*. 2023, Montesinos-López *et al*. 2024, Nikolaou *et al*. 2025). For *v* modalities, a base model $M^{\left(k\right)}$ outputs a score for sample *i*:


(3) $${\hat{p}}_{i}^{\left(k\right)}=M^{\left(k\right)}\left(x_{i}^{\left(k\right)}\right).$$

The final prediction is an aggregation: ${\hat{P}}_{i}=\text{Agg}\left({\hat{p}}_{i}^{\left(1\right)},\ldots ,{\hat{p}}_{i}^{\left(V\right)}\right)$.

**Figure 3:**
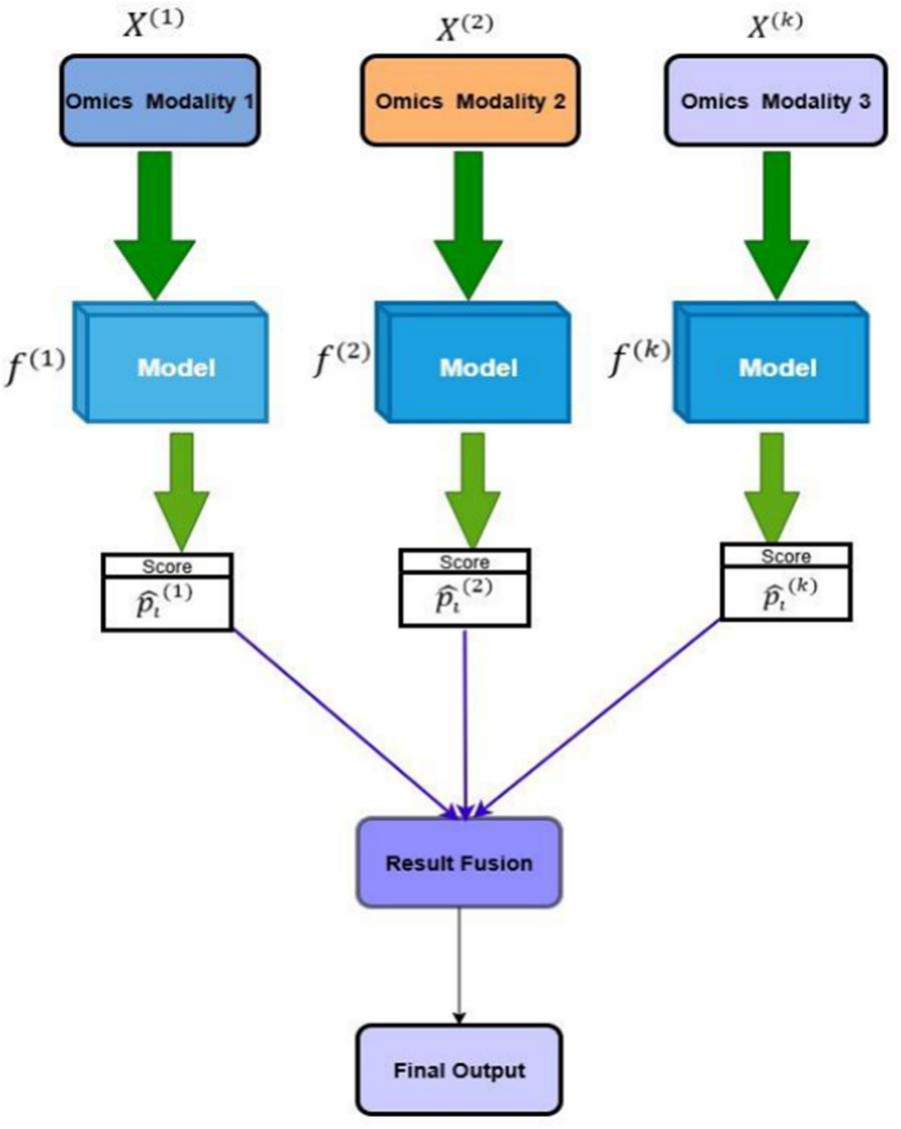
Late-fusion (decision-level) architecture for multi-omics integration. Each omics modality $X^{\left(1\right)},X^{\left(2\right)},\ldots ,X^{\left(k\right)}$ is processed by a dedicated model $f^{\left(1\right)},f^{\left(2\right)},\ldots ,f^{\left(k\right)}$ that produces a modality-specific score ${\hat{p}}_{i}^{\left(1\right)},{\hat{p}}_{i}^{\left(2\right)},\ldots ,{\hat{p}}_{i}^{\left(k\right)}$. These scores are then combined by a result fusion step to produce the final output. This architecture maximizes per-modality interpretability but may not capture synergistic cross-modal signals.

While maximizing interpretability (Figure 3), this approach may miss the synergistic effects arising from coordinated molecular processes (Carrillo-Perez *et al*. 2022, Salvi *et al*. 2024). A comparison of fusion stages is detailed by Montesinos-López *et al*. (2024).

### Linear projection-based methods

Linear projection methods, such as Principal Component Analysis (PCA) and Non-negative Matrix Factorization (NMF), are favoured for their interpretability and mathematical clarity in analysing high-dimensional biological data (Heo *et al*. 2021, Hernández-Lemus and Ochoa 2024). These techniques distill complex omics datasets into latent components to uncover dominant patterns of variation shared across molecular layers (Argelaguet *et al*. 2018, Mercadié *et al*. 2025).

**Figure 4:**
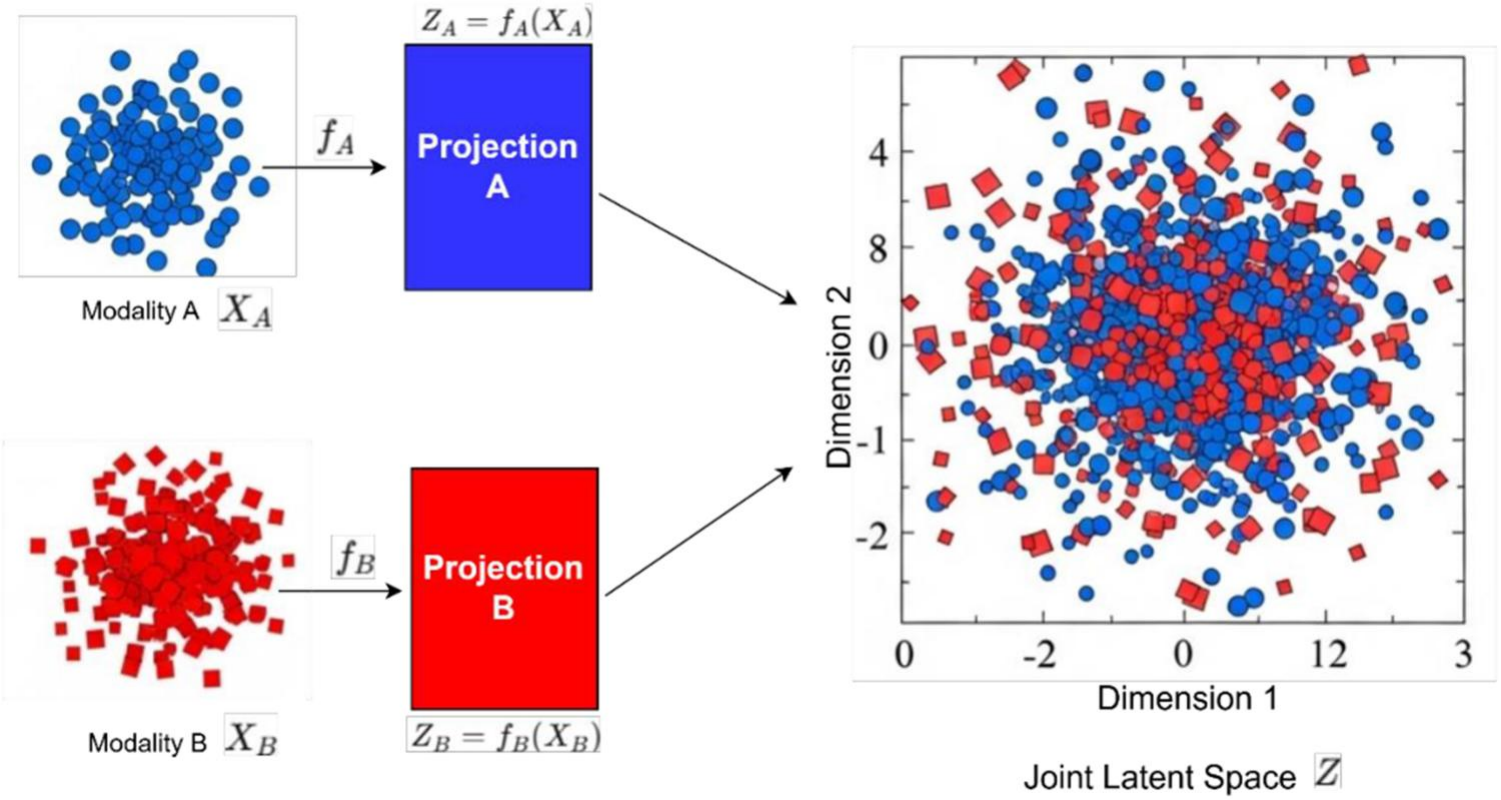
Projection-based integration into a joint latent space. Modality A ($X_{A}$) and Modality B ($X_{B}$) are independently mapped by view-specific projection functions $f_{A}$ and $f_{B}$ to low-dimensional embeddings $Z_{A}=f_{A}(X_{A})$ and $Z_{B}=f_{B}(X_{B})$. The resulting embeddings are aligned in a shared joint latent space *Z*, providing unified sample coordinates for downstream tasks such as clustering or survival analysis.

Formally, views $X^{\left(1\right)}$ and $X^{\left(2\right)}$ measured on *N* samples are mapped by view-specific projections $W^{\left(1\right)}$ and $W^{\left(2\right)}$ to low-dimensional embeddings $z^{\left(1\right)}=X^{\left(1\right)}W^{\left(1\right)}$ and $z^{\left(2\right)}=X^{\left(2\right)}W^{\left(2\right)}$. These embeddings are aligned into a joint latent space *Z* providing shared sample coordinates (Figure 4).

As detailed in Table 1, linear projection functions either as a primary integrator or a staging step. In intermediate fusion, view-specific embeddings are aligned into a common space *Z* for downstream tasks (Yang and Li 2025). Conversely, early fusion entails standardizing and concatenating features $X=[X^{\left(1\right)},X^{\left(2\right)}]$ prior to projection, yielding a single coordinate system (Mafata *et al*. 2022). Yang and Li (2025) exemplify projection as a central mechanism, estimating a shared latent space from partially observed data to enable clustering and survival analysis without concatenation (Yinghua *et al*. 2025). In contrast, studies such as Bai and Zhang (2023), Chalepaki *et al*. (2025), Du *et al*. (2025), and Zhu *et al*. (2025) utilize projection for early fusion, where PCA extracts components to feed downstream models like Graph Neural Networks (GNNs).

**Table 1:** Linear projection-based methods for multi-omics integration, detailing the specific technique used, its role in the pipeline, associated dimensionality reduction strategies, and omics types covered.

Reference	Projection Technique	Role in Pipeline	Method Name	Omics Types
Chalepaki *et al.* (2025)	Fold-change filtering, correlation scoring	Signature scoring and comparative analysis	Treg Signature Comparative Analysis	Transcriptomics, CNV, Methylation, SNV, Drug Sensitivity
Qin *et al.* (2023)	Correlation-based gene set scoring	Prognostic modelling and immune correlation	Cuproptosis Activity Scoring	Transcriptomics, CNV, Methylation
Luo *et al.* (2020)	Fold-change filtering, PCA (for drug sensitivity stratification)	Early fusion for downstream correlation	BMP Dysregulation Mapping	Transcriptomics, Epigenomics, Pharmacogenomics
Li *et al.* (2022)	Feature selection via Lasso and correlation	Preprocessing for ensemble learning	Ensemble Driver Gene Classification	Mutation, Epigenetics, Genomics, Phenotypic
Chen *et al.* (2024)	Fold-change filtering, PCA for immune stratification	Early fusion for survival modelling	HDAC Profiling	Transcriptomics, Epigenomics, Proteomics, Clinical
Moravveji *et al.* (2022)	Network propagation post-correlation filtering	Projection used for scoring and enrichment	GI Cancer Driver Discovery	Transcriptomics, Epigenomics, Genomics
Scott *et al.* (2021)	Meta-analysis (Hedges’ *g*), PCA for cross-platform harmonization	Projection for biomarker validation across modalities	KRT8 Biomarker Validation	Methylation, Bulk/Single-cell Transcriptomics, Proteomics, ELISA

The choice between intermediate fusion and early-stage projection reflects task demands. Intermediate fusion (Yang and Li 2025) creates compact, modality-aware embeddings suitable for missing data, whereas early-fusion PCA provides tractable input geometry for models like GNNs or Cox regressions. Bai and Zhang (2023) demonstrate this by constructing sample–sample graphs in PCA space to guide classification across 33 cancer types.

While projection offers conservative, noise-tolerant dimensionality reduction, strategies differ in handling missingness. Yang and Li (2025) directly accommodate missing assays, while early-fusion PCA generally requires harmonization or imputation followed by standardization to prevent variance domination (Bai and Zhang 2023, Chalepaki *et al*. 2025).

Interpretability remains a core strength of this family. Linear mappings expose feature loadings and sample scores for statistical analysis. Yang and Li (2025) report clustering performance via Adjusted Rand Index (ARI 0.8697) and correlate factors with clinical outcomes. When projection precedes modelling, interpretability is distributed; Bai and Zhang (2023) use PCA-derived neighbourhoods to inform attention weights, achieving high classification accuracy (0.9727).

Similarly, Du *et al*. (2025) and Zhu *et al*. (2025) leverage principal scores in survival modelling. However, trade-offs exist: early fusion simplifies analysis but often necessitates sample exclusion (Cantini *et al*. 2021), while intermediate projection accommodates missing data but may emphasize shared signals over modality-specific variation.

### Kernel-Based methods

Kernel-based integration (within the scope of this review) is represented by a single study (Huang and Kuan 2024) using a late-fusion approach centred on sample–sample similarity, building on kernel-learning integrative clustering ideas (Cabassi and Kirk 2020). Each omics modality is first reduced to an N×N kernel matrix in the form of a consensus matrix derived from resampling-based consensus clustering (Coleman *et al*. 2022, Gan and Allen 2022, Huang and Kuan 2024), before integration is performed through a convex combination.

The final integrated representation $K_{\text{integrated}}=\sum_{v}\alpha_{v}K^{\left(v\right)}$, where $\alpha_{v}\geq 0$ and $\sum \alpha_{v}=1$, reflects a weighted consensus across views (Cabassi and Kirk 2020, Huang and Kuan 2024). Related kernel-fusion strategies for multi-omics subtyping have also appeared in recent work (Feng *et al*. 2021, Rintala and Fortino 2024, Cao *et al*. 2025).

This strategy minimizes the risk of noisier or less informative assays dominating the output by deferring fusion until after each view has been converted into a consensus-based similarity representation and can be downweighted (Cabassi and Kirk 2020, Huang and Kuan 2024). Simulations and a pan-cancer TCGA application in Huang and Kuan (2024) show that weighting with consensus-based kernels can improve robustness and clustering agreement across modalities.

The underlying assumption is that biologically meaningful structure produces clearer within-cluster agreement (and lower between-cluster agreement) in the consensus kernels, and integration aims to preserve this signal (Huang and Kuan 2024). This aligns with pan-cancer goals of identifying coherent sample groupings supported by multiple assays rather than a single data type.

A key premise is that a set of weights $\alpha_{v}$ effectively summarizes each view’s contribution. If a modality offers noisier kernels, its weight is downregulated without discarding the view (Cabassi and Kirk 2020, Huang and Kuan 2024). Because integration targets sample-level geometry rather than feature-level mappings, cross-modality feature interactions are not explicitly modelled; biological interpretation occurs downstream after clusters are identified (Feng *et al*. 2021, Huang and Kuan 2024).

This design works at the level of sample–sample kernels, so it avoids the need to directly match features across heterogeneous measurement spaces (Cabassi and Kirk 2020, Cao *et al*. 2025). In practice, fusion is usually performed over a shared set of samples across modalities, and extensive missingness typically needs additional preprocessing decisions outside the kernel-fusion step itself. Modality imbalance is managed through the weighting scheme, while consensus-building steps such as resampling are used to promote robustness to perturbations within each view (Cabassi and Kirk 2020, Coleman *et al*. 2022, Gan and Allen 2022, Huang and Kuan 2024). Downstream validation proceeds via post hoc analysis: clusters from $K_{\text{integrated}}$ are compared against external labels and clinical endpoints, and may also be followed by pathway-level interpretation depending on the study (Feng *et al*. 2021, Huang and Kuan 2024, Rintala and Fortino 2024). The authors emphasize stability-based validation, such as clustering agreement and robustness under resampling, consistent with the method’s exploratory nature (Gan and Allen 2022, Huang and Kuan 2024).

By privileging neighbourhood structure, this approach prioritizes broad, cross-modal sample subtypes over fine-grained regulatory mechanisms. Generality is demonstrated through simulation and cancer multi-omics applications (Cao *et al*. 2025). This kernel-based strategy is well-matched to settings requiring stable, interpretable subtype maps, offering resistance to noisy data blocks while deferring feature attribution to downstream analysis. Evaluation metrics, including Adjusted Rand Index, are commonly used to assess agreement between derived partitions and reference labels or known subtype structure (Cabassi and Kirk 2020, Feng *et al*. 2021, Huang and Kuan 2024, Rintala and Fortino 2024).

### Probabilistic latent variable models

Probabilistic integration is a small part of the literature in this review, but it is useful because this method family makes the integration step explicit. The method does not rely on a sequence of preprocessing choices to create integration at the end. Instead, it starts by stating what the shared signal is meant to be—a latent structure or a set of important features—and how each omics view is supposed to relate to it (Argelaguet *et al*. 2020, González-Reymundez *et al*. 2022, Wang *et al*. 2023).

Because of that, these models are easier to compare in that they inform how the datasets are meant to be fused and what is done when there is missing data, instead of leaving those decisions to a string of preprocessing steps that can be hard to see or separate (Argelaguet *et al*. 2020, Gayoso *et al*. 2021, Park and Hauschild 2024).

In Table 2, the three papers fall into two families: a shared latent representation model that fuses assays through a common latent space (Yang and Li 2025), and feature-centred Bayesian models that fuse by turning cross-omics evidence into calibrated priors for variable selection (Bhattacharyya *et al*. 2024, 2022).

**Table 2:** Overview of probabilistic integration models reviewed in this study.

Study	Strategy Type	Model Formulation	Integration Method	Omics Types	Missing Data Handling	Inference & Uncertainty	Application/Key Metrics
Yang and Li (2025)	Shared latent representation	Latent variable model with Gaussian noise	Generalized probabilistic CCA	Multi-omics (transcriptomic, epigenomic)	Likelihood supports partial observations	Variational EM; posterior over latent *Z*	Shared latent structure; clustering; ARI, survival association
Bhattacharyya *et al.* (2024)	Feature-centred Bayesian	Hierarchical Bayesian regression; spike-and-slab priors on features	Feature inclusion probabilities	Multi-platform clinicogenomic	Missing handled by priors and evidence synthesis	MCMC posterior sampling	Feature selection; predictive performance; calibration diagnostics
Bhattacharyya *et al.* (2022)	Feature-centred Bayesian	Bayesian evidence synthesis; external priors	Cross-omics feature relevance	Multi-omics (mechanistic priors + outcomes)	Missing handled via prior support consistency	Posterior inference with simulations	Parsimonious feature panels; prediction accuracy; posterior stability

The table distinguishes shared latent-space and feature-centred Bayesian designs, and summarizes their probabilistic assumptions, integration mechanisms, omics coverage, treatment of missing data, uncertainty-handling approaches, and validation metrics.

### Shared Latent-Space models

This approach assumes that a smaller set of hidden variables captures the main biological patterns shared across all the omics layers. For each omics view $X_{m}$, the data are represented as:


(4) $$X_{m}\approx f_{m}(Z)+\epsilon_{m},$$

Where *Z* denotes latent factors common to all modalities, $f_{m}$ is a mapping specific to each omics type, and $\epsilon_{m}$ is random noise. In linear cases such as Yang and Li (2025) this takes the form $f_{m}(Z)=ZA_{m}^{⊤}$, with weight matrices learned from the data. Because the likelihood is defined even when some assays are missing, samples with incomplete observations can still contribute to estimating *Z*, yielding a compact latent representation that unifies the assays, emphasises signals recurring across layers, and quantifies uncertainty.

GPCCA assumes the main signal is a shared low-dimensional *Z* that is present across R≥2 modalities. Each modality is modelled as a linear mapping of *Z* plus Gaussian error, and the full model is built by stacking all modalities. The noise structure is explicit too: the error covariance is block-diagonal, so correlations are allowed within a modality but not across modalities.

Missing data is handled inside the model by deriving an EM algorithm under a Missing-At-Random (MAR) assumption and introducing an observation indicator matrix to separate observed from missing entries. Model robustness was tested when MAR is violated by simulating MNAR patterns, including modality-wise missingness, because it was argued that this can happen in biomedical data.

Upon evaluation, ARI/NMI was reported on a multi-modality digit dataset and shows GPCCA remains top (or near-top) under MCAR and MNAR missingness scenarios. For TCGA, Yang and Li (2025) analyzed 10 cancer types with three modalities (gene expression, methylation, miRNA), clustered embeddings with Louvain, and used survival differences as a proxy for cluster quality (log-rank and Cox LRT with BH correction).

### Feature-centered Bayesian models

The second probabilistic strategy does not embed all data into a shared space but directly evaluates which features are relevant for a given outcome using Bayesian selection mechanisms. For a feature *j*, an indicator variable $\gamma_{j}$ denotes whether it is predictive. Before seeing the data, the model assigns a prior probability $p(\gamma_{j})$, reflecting how plausible it is that the feature is relevant based on external knowledge or cross-omics support—for example, a gene already implicated in cancer pathways or supported by mechanistic studies may receive a higher prior probability than one with no prior evidence.

After observing the data, this belief is updated using Bayes’ rule:


(5) $$p\left(\gamma_{j}|\text{data}\right)\propto p\left(\text{data}|\gamma_{j}\right)p(\gamma_{j}),$$

Where $p(\text{data}|\gamma_{j})$ is the likelihood of seeing the observed data if feature *j* is truly predictive, and $p(\gamma_{j}|\text{data})$ is the posterior inclusion probability combining prior knowledge with observed evidence, providing a calibrated probability that the feature is genuinely associated with the outcome.

The fiBAG (Bhattacharyya *et al*. 2024) and BaySyn (Bhattacharyya *et al*. 2022) do not fuse data by learning one shared *Z*. Instead, they build evidence first, then use that evidence to calibrate spike-and-slab selection in an outcome model.fiBAG is explicit about the two layers: Gaussian process mechanistic models generate functional evidence (via Bayes factors), and those evidences are mapped to a calibrated spike-and-slab prior to guide selection and effect estimation for patient outcomes. They benchmark selection using metrics like AUC and MCC, and run a TCGA pan-cancer analysis across four cancer groups covering 14 cancers, focusing on stemness and survival.

BaySyn makes the same “evidence to calibrated selection” move, but across systems (cell lines and patients). It uses additive GP models to infer mechanistic evidence for driver genes and uses that evidence to calibrate Bayesian variable selection in drug response models, which was thereafter applied to CCLE and TCGA in pan-gynecological cancers, reporting enrichment of mechanistic evidence in KEGG gene sets, and shows calibrated outcome models make more discoveries than uncalibrated counterparts under the same error-control thresholds.

### Deep learning models

Deep learning models form another important family of multi-omics integration approaches. Unlike the probabilistic frameworks discussed in Section 3.4, they do not define explicit likelihoods and priors; instead, they rely on neural architectures to capture complex, nonlinear relationships across heterogeneous assays. The way these models integrate information is driven by architectural design choices, often described in terms of the “fusion point”—whether features are combined at the input level, at intermediate latent representations, or only at the prediction stage. These choices reflect underlying assumptions about how biological signals are shared, how missing or imbalanced data should be handled, and how results can be interpreted and generalized.

A common strategy is to use modality-specific encoders, where each omics matrix $X_{m}$ is transformed into a lower-dimensional representation $h_{m}=f_{m}(X_{m})$, and these representations are then aggregated into a joint embedding $Z=g(h_{1},h_{2},\ldots ,h_{M})$.

Deep learning shows up more as a set of design choices about where fusion happens. Early vs representation-level vs interaction-level fusion ends up setting the ground rules: what counts as shared signal, how much the model can lean on one modality, and what breaks first when assays are missing. This is not just theory; benchmarks in cancer multi-omics show that integration gains are not automatic, and robustness (noise/missingness) is often the differentiator (Cantini *et al*. 2021, Wissel *et al*. 2023, 2025).

Guo *et al*. (2022) used early fusion in a pathway-focused way by concentrating on the Notch pathway: they integrate mRNA, CNV, methylation, and miRNA features for core Notch genes, normalise, then use a denoising autoencoder to compress the data before Cox modelling and C-index evaluation. The assumption is that “cross-omics patterns are learnable once everything is in one space.” Missing data is handled pragmatically (median fill, remove high-missingness samples, then standardise). Interpretability is helped because the input is already biologically scoped (Notch genes/targets and related RNA networks), not a genome-wide black box (Guo *et al*. 2022).

A second family is representation-level fusion, which learns a representation per modality first, then fuses them. MOSAE fits here. It uses one supervised autoencoder per omics and fuses the latent vectors by averaging (Tan *et al*. 2020). The idea is that averaging forces the same latent coordinates to line up across omics, so the shared signal is whatever remains consistent across modalities. This comes with its own drawback in terms of missingness: for each endpoint, only the samples with all four omics present were used (Tan *et al*. 2020).

MFHCC is in the same category of model that first separates the encoders, then combines them afterwards. Notwithstanding, it treats multi-omics as multi-view, by using an autoencoder per view, then adds hierarchical contrastive learning and a clustering module so the learned structure is consistent across views (Jiang *et al*. 2023b). That is a stronger signal assumption than MOSAE: it is not just that modalities are useful, it is that there is a clusterable pattern that more than one omics layer should support. Missingness is again handled mainly by cohort choice (patients with jointly available multi-omics data) Jiang *et al*. 2023b). More recent models in the same direction make the “separate noise vs consistent signal” goal explicit using VAE + contrastive + attention style components (Cai and Wang 2024).

Then there is interaction-level fusion, which matters in pan-cancer because it does not just merge representations, but rather models cross-modal interactions. Chen *et al*. (2022) build unimodal representations for WSI and molecular profiles, then fuse them using Kronecker Product Fusion (so interactions are part of the representation), with a gating step that controls how much each modality contributes. This interaction-fusion idea builds on earlier multimodal histology-genomics fusion work. Here, missingness is mostly about paired availability: they train on matched WSI–molecular data across 14 TCGA cancer types and describe pairing criteria where needed. Interpretability is built in: WSI attention heatmaps plus molecular attributions (PORPOISE) (Chen *et al*. 2022).

However, not all “deep learning integration” here is multi-omics in the strict sense. Wang *et al*. (2024) instead integrate biomarker “levels” inside a gene-expression input using self-attention, training a multi-task model for anti-PD1/PD-L1 response; because labelled ICI outcomes are limited, they use proxy training (TMB and PD-L1) and validate on external ICI-treated cohorts.

More broadly, in this deep-learning family, what matters is where fusion happens, since that sets the trade-offs and benchmarks show extra modalities can improve performance or degrade it when noise and missingness are not handled well (Wissel *et al*. 2023, 2025).

### Graph-based and hybrid models

Graph-based models adopt a relational perspective, prioritizing patient or gene relationships over raw feature matrices. These approaches build networks where edges capture similarity or biological associations, subsequently propagating information over these structures.

A common approach defines similarity graphs where edge weights $W_{ij}=\text{similarity}(x_{i},x_{j})$ represent correlations or distances. Chierici *et al*. (2020) employ distance-based networks for each omics type, iteratively diffusing them into a consensus network. This late fusion strategy amplifies cross-omics agreement while muting spurious edges, with robustness confirmed by high Matthews correlation coefficients.

Alternatively, early integration strategies construct graphs on unified feature sets. Bai and Zhang (2023) apply PCA to harmonized multi-omics data to build patient–patient graphs for a graph attention network (GAT) classifying 33 cancer types. Similarly, MetaGXplore (Jiang *et al*. 2024) utilizes Graph Convolutional Networks (GCNs). The core operation updates node representations based on neighbors:


(6) $$H^{\left(l+1\right)}=\sigma \left(\hat{A}H^{\left(l\right)}W^{\left(l\right)}\right),$$

Where *H* represents integrated features and $\hat{A}$ is the adjacency matrix. These methods assume local sample relationships are predictive, using propagation to uncover latent metastatic structures.

Hybrid strategies incorporate external knowledge or operational constraints. Moravveji *et al*. (2022) diffuse composite scores over fixed KEGG pathways to highlight gastrointestinal cancer subnetworks, focusing on interpretable biological modules. Li *et al*. (2022) combine early fusion with classical models to prioritize driver genes, emphasizing reproducibility. Addressing privacy, Mohammed *et al*. (2023) implement federated learning, where local autoencoders compress representations for central aggregation without sharing raw data.

Assumptions and missing data handling vary by design. Diffusion methods (Chierici *et al*. 2020) average available omics graphs, mitigating incomplete views upstream. Learned-graph models (Bai and Zhang 2023, Jiang *et al*. 2023b) rely on distinct early integration steps, necessitating harmonization. Federated designs (Mohammed *et al*. 2023) manage missingness locally. Interpretability aligns with architecture: consensus graphs reveal stable clusters, pathway diffusion validates biological modules, and attention weights (Moravveji *et al*. 2022, Bai and Zhang 2023) elucidate influential patient relationships. These strategies distinctively balance biological insight with data constraints through diverse fusion and propagation logic.

## Results and comparative discussionnn

**Figure 5:**
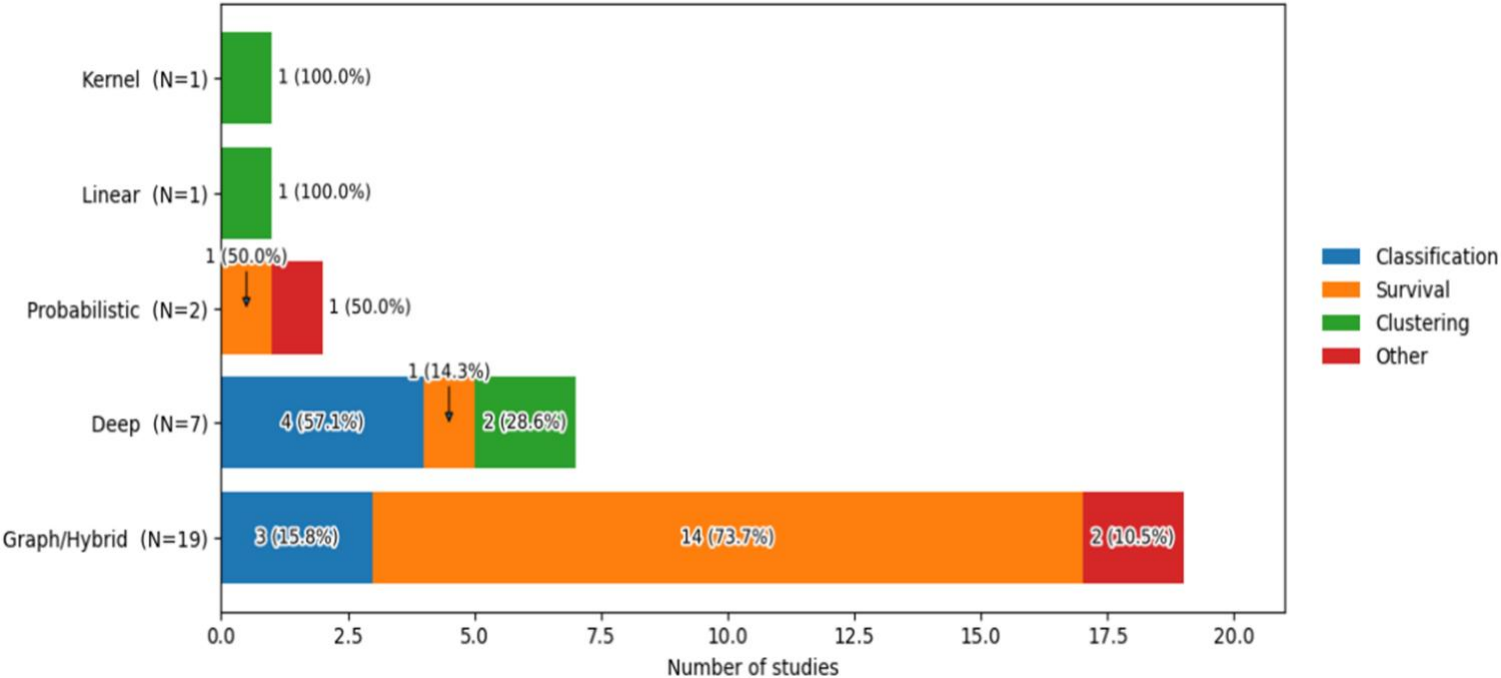
Task composition by method family (100% stacked bar chart). Each bar shows the number of studies per method family coloured by analytical task: classification (blue), survival (orange), clustering (green), and other (red). Graph/Hybrid methods (N=19) are heavily skewed toward survival prediction (14 studies; 73.7%), while Deep Learning (N=7) spans classification, survival, and other tasks. Kernel (N=1) and Linear (N=1) methods both focus exclusively on clustering, and Probabilistic methods (N=2) split evenly between clustering and survival.

The reviewed corpus (n=30) is dominated by graph-hybrid methods (n=19) and deep learning (n=7), with a secondary presence of probabilistic latent variable models (LVMs), linear projections, and kernel-based approaches. This distribution, primarily oriented toward survival analysis (16/30) and classification (n=7), reflects a clear field-wide shift toward patient-level prognostic modeling (Figure 5).

Distinct method-task pairing patterns emerge across studies: linear and kernel methods focus exclusively on clustering, while deep learning exhibits high architectural flexibility across classification and survival tasks. In contrast, graph-hybrid methods are heavily skewed toward survival prediction (73.7%), suggesting a preference for graph-augmented designs in pan-cancer prognostic settings.

Fusion strategies largely track these methodological families. Deep learning spans early (43%), intermediate (29%), and late (29%) fusion, whereas graph-hybrid methods prioritize early-to-intermediate stages to facilitate pre-learning graph construction (Figure 5). Analytical goals further dictate fusion timing; early and intermediate fusion stabilize inputs for downstream graph classifiers or latent space estimation from partially observed assays.

Conversely, late fusion—prevalent in kernel and diffusion methods—is preferred for recovering robust sample partitions where relational consistency supersedes feature-level integration.

These design choices are underpinned by specific biological and technical assumptions. Projection models assume low-dimensional structural axes, whereas shared-space models and federated approaches are better suited for handling missing data and incomplete assays. Interpretability also scales with architecture: projection methods offer transparent factor loadings, probabilistic models quantify uncertainty via posterior probabilities, and deep learning increasingly utilizes attention weights or post-hoc profiling.

Graph-based designs uniquely reveal neighbourhood saliency and pathway subnetworks, capturing relational hypotheses often missed by feature-wise integrators.

Ultimately, three core trade-offs emerge: early/intermediate fusion via PCA stabilizes high-dimensional data, while shared latent spaces better preserve partially observed samples; late fusion excels in robust subtype detection, whereas probabilistic methods favour interpretable feature panels; and deep architectures balance performance with readability, complemented by graph-based methods for relational modelling. Multi-omic integration must therefore be treated as a deliberate design decision rather than generic preprocessing that dictates which biological structures are preserved or abstracted.

## Conclusion and future directions

This review examined five distinct families of multi-omics integration methods in pan-cancer studies: linear projection, kernel-based approaches, probabilistic integration models, deep learning architectures, and graph-based or hybrid systems. Rather than evaluating these models in isolation, the analysis emphasized how each family encodes biological assumptions, manages missingness, and aligns fusion strategy with analytical objectives.

Synthesis reveals no universal optimal method; instead, a co-evolution exists between integration design and task framing. Linear and kernel-based methods provide interpretability and stability, serving as foundational steps for discovery or subtype recovery. Probabilistic models foreground transparency and calibrated uncertainty, suiting tasks requiring rigorous feature attribution. Deep learning offers architectural flexibility and scale but demands careful harmonization. Graph-based and hybrid methods distinguish themselves by embedding relational structure directly into the modelling process, exposing patterns opaque to feature-level models.

Methodological choices are most effective when tightly coupled to data structure and biological inquiry. Early or intermediate fusion stabilizes inputs for complex learners, whereas late fusion emphasizes cross-modality coherence at the relational level. Integration in the pan-cancer paradigm is a modelling decision space that requires pairing technical execution with scientific intent.

Future progress depends on refining the match between integration strategies, biological questions, and the constraints of multi-cohort cancer data. The central challenge in pan-cancer multi-omics integration lies in aligning methods with data realities and analytic objectives. Translational pipelines require models that explicitly accommodate incomplete assays and provide interpretable outputs, while discovery-driven settings must prioritize partition robustness. Modular, stacking, or federated strategies offer pragmatic pathways as cohorts evolve.

## Data Availability

This article is a systematic review. No new data were generated or analysed. All data discussed are derived from previously published studies identified through the PRISMA-guided search described in the Methods section. The full list of included studies and their associated data sources are available in the references.
